# Metabolomic Studies of Oral Biofilm, Oral Cancer, and Beyond

**DOI:** 10.3390/ijms17060870

**Published:** 2016-06-02

**Authors:** Jumpei Washio, Nobuhiro Takahashi

**Affiliations:** Division of Oral Ecology and Biochemistry, Graduate School of Dentistry, Tohoku University, 4-1 Seiryo-machi, Aoba-ku, Sendai 980-8575, Japan; nobu-t@dent.tohoku.ac.jp

**Keywords:** metabolomics, oral biofilm, oral cancer, metabolism

## Abstract

Oral diseases are known to be closely associated with oral biofilm metabolism, while cancer tissue is reported to possess specific metabolism such as the ‘Warburg effect’. Metabolomics might be a useful method for clarifying the whole metabolic systems that operate in oral biofilm and oral cancer, however, technical limitations have hampered such research. Fortunately, metabolomics techniques have developed rapidly in the past decade, which has helped to solve these difficulties. *In vivo* metabolomic analyses of the oral biofilm have produced various findings. Some of these findings agreed with the *in vitro* results obtained in conventional metabolic studies using representative oral bacteria, while others differed markedly from them. Metabolomic analyses of oral cancer tissue not only revealed differences between metabolomic profiles of cancer and normal tissue, but have also suggested a specific metabolic system operates in oral cancer tissue. Saliva contains a variety of metabolites, some of which might be associated with oral or systemic disease; therefore, metabolomics analysis of saliva could be useful for identifying disease-specific biomarkers. Metabolomic analyses of the oral biofilm, oral cancer, and saliva could contribute to the development of accurate diagnostic, techniques, safe and effective treatments, and preventive strategies for oral and systemic diseases.

## 1. Introduction

### 1.1. What Is Metabolomics?

Metabolomics is a relatively new form of omics research. Living cells contain many metabolites, which are derived from various metabolic activities. These metabolites are the final products of cellular biochemical processes, including gene transcription, mRNA translation, protein synthesis, and metabolic enzymatic reactions. The comprehensive identification and quantification of these metabolites is called “metabolomics”. Metabolomics is essential to clarify cellular function.

In the past decade, metabolomics techniques have advanced markedly. Until recently, it was technically difficult to comprehensively and simultaneously analyze numerous metabolites, especially small ionic metabolites, such as phosphorylated sugars, carbonic acids, and amino acids, in the same sample. However, in the 2000s, new techniques, such as capillary electrophoresis (CE) and a time-of-flight mass spectrometer (TOFMS), were developed. CE consists of a capillary (diameter: 100 µm or less) and an electrode. The samples are injected into the capillary and a high voltage is applied to both ends of the capillary, causing small ionic metabolites in samples to move to the cathode or anode. The ionic radius and the electric charge of each metabolite are different, the movement velocities of each metabolites in the capillary is also different; therefore, the ionic metabolites in the sample can be separated in the capillary. The separation ability of CE is very excellent; the liquid chromatography cannot separate the metabolites having the same mass, while CE can separate them by a slight difference of the electric charge. On the other hand, TOFMS, one of the devices for mass spectrometry, can measure the mass of ionic metabolites very precisely by measuring the flight time. By connecting these two apparatuses, these techniques have made it possible to separate small ionic molecules and to measure their masses precisely. The mass-to-charge ratio (*m*/*z*) and the precise mass of the metabolite acquired through the measurement with CE-TOFMS, enable us to identify the metabolite [1,2]. In addition, they only require small samples. Since then, CE-TOFMS has been used in various areas of metabolomics research, particularly for studies into central carbon metabolism including the Embden-Meyerhof-Parnas (EMP) pathway, the pentose phosphate (PP) pathway, and the tri-carbonic acid (TCA) cycle, as well as amino acid metabolism-related pathways [3,4,5,6,7].

### 1.2. Advantages of CE-TOFMS-Based Metabolomics for Oral Biofilm and Oral Cancer Research

Most previous bacteriological studies of the oral biofilm have asked “What are they?”, that is, what kinds of bacteria are found in the oral biofilm or at the oral disease sites [8,9,10,11,12]. However, “What are they doing?”, that is, the functional metabolic properties of the oral biofilm or the bacteria within it, have not been investigated sufficiently [13]. Bacteria produce various metabolites. The enzymatic activities involved in these metabolic reactions and the resultant metabolic products might directly contribute to oral diseases [13]. It is known that organic acids, such as lactic acid, produced by bacterial sugar metabolism through the EMP pathway, initiate dental caries [14], while the short-chain fatty acids, ammonia, and sulfur compounds produced through the bacterial metabolism of protein and amino acids initiate periodontal disease [15,16,17].

A method for quantifying the concentrations of the metabolic intermediates that participate in the EMP pathway in human red blood cells based on photometry-coupled enzymatic reactions involving a purified glycolytic enzyme was developed [18]. This method has since been used to investigate the metabolite profile and metabolic regulation of the EMP pathway in oral bacteria, such as oral *Streptococcus* and *Actinomyces* [19,20,21,22,23,24,25,26,27,28,29], and the inhibitory effects of fluoride and xylitol on bacterial sugar metabolism [30,31,32,33,34,35]. However, because of technical limitations, this conventional method has only been used to target the metabolites in the EMP pathway.

**Figure 1:**
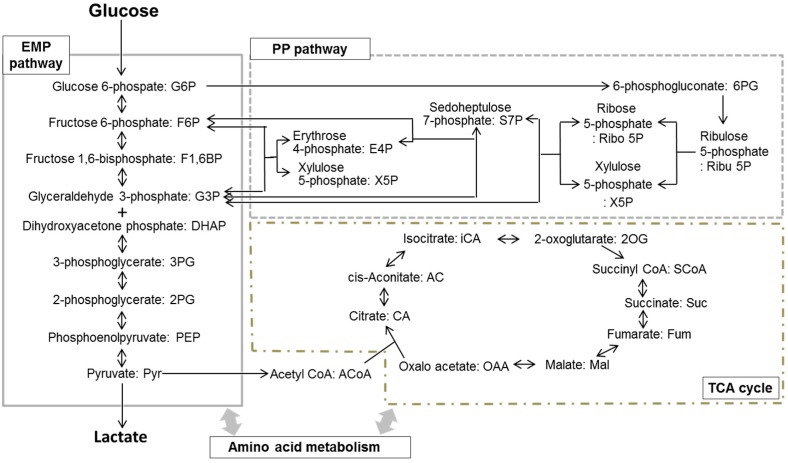
Schema of the central carbon metabolism pathway, including the Embden-Meyerhof-Parnas-pathway (EMP pathway), the pentose-phosphate pathway (PP pathway), and the tricarboxylic acid cycle (TCA cycle).

Bacterial sugar metabolism is known to be related to other carbon metabolic pathways, including the PP pathway and the TCA cycle, as shown in Figure 1. Most periodontitis-related bacteria, which form part of the oral biofilm, utilize not only sugars, but also amino acids, as energy and carbon substrates [36]. Thus, the comprehensive detection of the various metabolites related to amino acid metabolism is also essential for clarifying the metabolic system that operates in the entire oral biofilm. In addition, the conventional method requires quite large samples, even for analyses of single metabolites; therefore, it is difficult to measure various metabolites in the same sample. In particular, clinical specimen, such as oral biofilm samples, are too small to be analyzed using this method. Due to such technical limitations, previous studies of biofilm metabolism have been based on *in vitro* techniques involving the culturing of a single bacterium, whereas the oral biofilm is known to be composed of over 500 kinds of bacteria. As described above, CE-TOFMS can detect various metabolites and only requires a small sample. Therefore, it can be used for metabolomic research involving clinical specimens, such as oral biofilm samples.

On the other hands, it is considered that cancer cells possess different metabolic properties from the normal cells. The most famous of phenomena is the ‘Warburg effect’ [37]. The Warburg effect, which involves lactate production during glucose metabolism even in the presence of abundant oxygen, is a common metabolic characteristic of cancer cells, suggesting that the EMP pathway mainly functions under aerobic conditions (aerobic glycolysis). Disordered metabolism is considered to contribute to cancer progression and malignancy. Therefore, increasing our understanding of the metabolic profile of cancer cells might help to elucidate the malignant changes that occur in cancer and aid the development of new approaches to cancer treatment. Similar to the oral biofilm, cancer cells also utilize various substrates as energy and carbon sources [38] and clinical oral cancer specimens tend to be quite limited in size. Thus, CE-TOFMS-based metabolomics analysis is also useful for aiding research into the metabolic processes that occur in cancer cells/tissues.

In this article, we would like to review novel metabolomics studies related to oral diseases and compare them with the findings of our recent studies.

## 2. Metabolomics Research into the Oral Biofilm

The first CE-TOFMS-based metabolomics study of the oral biofilm was performed at our laboratory [39,40]. The metabolomic profiles of the oral biofilm and representative oral bacteria before and after the addition of glucose were analyzed and compared [39]. The effects of fluoride and xylitol, which are caries-preventive reagents that are widely used around the world, on oral biofilm metabolism were also investigated using a metabolomic approach [40].

### 2.1. Sample Preparation for Metabolomic Analysis and the Study Method

In these studies [39,40], samples of supragingival plaque from health volunteers were used as the oral biofilm samples. The supragingival plaque was collected using sterilized toothpicks before, and 10 min after, rinsing with 10 mL of 10 mM glucose or a mixture of 10 mM xylitol plus 10 mM glucose for 60 s. In the fluoride trial, pre-rinsing was performed with 10 mL sodium fluoride (225 or 900 ppm F) for 60 s, and the samples were collected before and 10 min after the rinse with 10 mL of 10 mM glucose. For avoiding the effect of the daily variation of oral biofilm, we decided a timing to collect a sample at two hours after the last meal. The collected samples were weighed with an electronic scale, and mixed with ice-cold methanol immediately and sonicated (30 s) for extraction of metabolites. Furthermore, chloroform and milli-Q were added and vortexed for removing the materials, such as phospholipids, that may interfere with CE-TOFMS analysis. The aqueous layer was filtrated through an ultra-filtration membrane to remove proteins, and the filtrate was assessed using CE-TOFMS. Most of the metabolites involved in the EMP pathway, the PP pathway and the TCA cycle (Figure 1) were identified and quantified from the acquired raw data.

In addition, further experiments were performed using *Streptococcus mutans* NCTC 10449, *Streptococcus sanguinis* ATCC 10556, *Actinomyces oris* WVU 627, and *Actinomyces naeslundii* ATCC 12104 as representative bacteria [39]. The cells were collected using a membrane filter (pore size, 0.4 μm; Millipore) before and 10 min after the addition of glucose (final concentration; 10 mM) to the bacterial cell suspensions. Then, the collected bacterial cells were pre-treated, and analyzed in a similar manner to the oral biofilm samples.

### 2.2. Metabolomics of the Oral Biofilm and a Comparison with the Findings for Representative Oral Bacteria

The EMP pathway, the PP pathway, and the TCA cycle were detected and identified (Table 1), even when only a very small amount (less than 10 mg) of the oral biofilm was analyzed, suggesting that this method is suitable for metabolomic research into the oral biofilm. In addition, glucose rinsing resulted in marked changes in the samples’ metabolite profiles. These results indicated for the first time that the main carbon metabolic pathways, such as the EMP pathway, the PP pathway, and the TCA cycle (Figure 1), are in operation in the oral biofilm *in vivo*. In particular, increased levels of glucose 6-phosphate (G6P), fructose 6-phosphate (F6P), and fructose 1,6-bisphosphate (F1,6BP), and decreased levels of 3-phosphoglycerate (3PG) and phosphoenolpyruvate (PEP) were observed after the glucose rinsing, suggesting that the PEP-dependent sugar phosphotransferase system plays a role in glucose uptake in the oral biofilm. Some other metabolites, such as erythrose 4-phosphate (E4P), iso-citrate (iCA), and *cis*-aconitate (AC), were not detected or were only detected at low levels, suggesting that the metabolic pathways associated with these metabolites do not function at high levels in the oral biofilm.

As for the metabolomic profiles of the representative oral bacteria, all of the targeted EMP pathway, PP pathway, and TCA cycle metabolites were detected. In *Streptococcus mutans* and *Actinomyces naeslundii*, the increased levels of G6P, F6P, and F1, 6BP, and reduced levels of 3PG and PEP were observed after the addition of glucose, suggesting that the PEP-dependent sugar phosphotransferase system and glycolysis occur in these bacterial species. Furthermore, the increases in the levels of 6-phosphogluconate (6PG), ribose 5-phosphate (Ribo5P), and sedoheptulose 7-phosphate (S7P) seen after the addition of glucose indicated that the PP pathway also functions along with glucose metabolism in these bacteria. The concentrations of the TCA cycle metabolites changed after the addition of glucose in a bacterial species-dependent manner, suggesting that the entire, or part of the, TCA cycle is in operation in all of the examined bacteria.

In comparisons between the metabolomic profiles of the oral biofilm and representative oral bacteria, PEP-dependent sugar phosphotransferase system activity was observed in both the oral biofilm and some of the oral bacteria. Furthermore, increased levels of PP pathway metabolites were detected in the oral biofilm and some the oral bacteria during glycolysis. The PP pathway is known to supply NADPH for fatty acid synthesis and pentose phosphates for nucleotide synthesis. It was suggested that both energy and raw materials for cell growth are produced through the metabolism of glucose in the oral biofilm *in vivo*. The reduced amounts of succinate, fumarate, and malate (TCA cycle intermediates) detected in the oral biofilm, resembled the changes seen in *Actinomyces naeslundii.* Thus, the metabolite profile in the oral biofilm exhibited similar characteristics to those of *Streptococcus* and *Actinomyces* [41,42].

### 2.3. Effects of Fluoride and Xylitol on Metabolism in the Oral Biofilm

**Figure 2:**
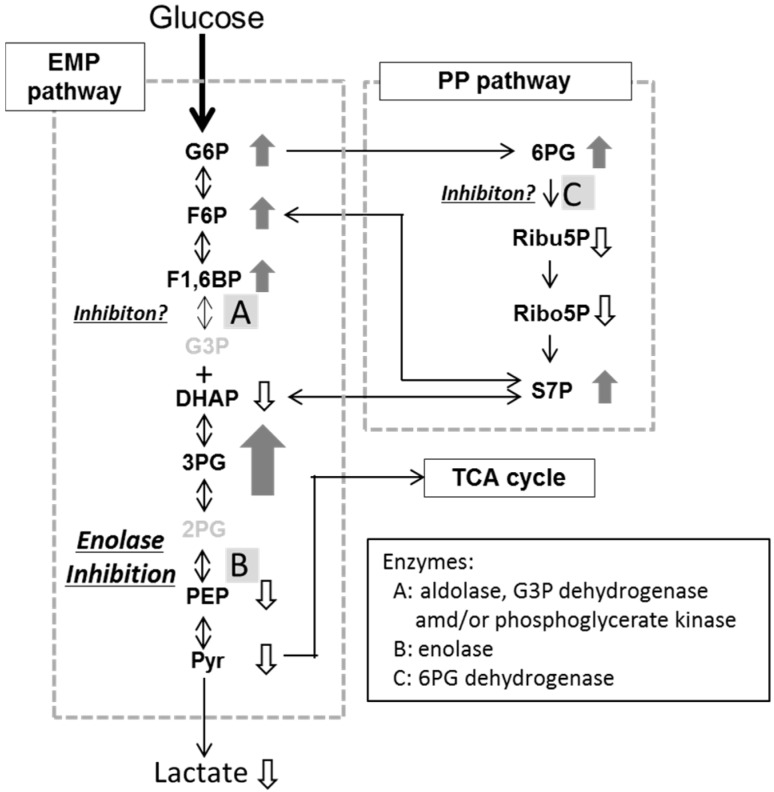
Schema of the effects of fluoride on the central carbon metabolic pathway. Small arrows with metabolite names, significant increases (upward arrow) or reductions (downward arrow) in the levels of the named metabolites. See Figure 1 for an explanation of the metabolite abbreviations.

Fluoride and xylitol are used as caries-preventive reagents around the world. Fluoride is known to inhibit bacterial acid production *in vitro* [43,44,45] and plaque acid production *in vivo* [46,47]. Enolase, an enzyme that is involved in the EMP pathway, is considered to be the target of fluoride inhibition [48,49]. The addition of fluoride to a cell suspension of oral streptococci, including *Streptococcus mutans*, inhibited enolase and resulted in the intracellular accumulation of 3PG, 2-phosphoglycerate (2PG), and PEP [31,34]. In the present *in vivo* trials, the addition of fluoride to the oral biofilm resulted in increased levels of G6P, F6P, F1, 6BP, 3PG, 6PG, and S7P, and lower levels of DHAP, PEP, and pyruvate. Lactate production was significantly inhibited by fluoride rinsing (Table 2). The accumulation of 3PG along with decreased lactate production was indicative of *in vivo* enolase inhibition (Figure 2). The increase in the amounts of G6P, F6P, and F1, 6-BP, and the slight reduction in the amount of DHAP were suggestive of the inhibition of aldolase, glyceraldehyde 3-phosphate dehydrogenase, and/or phosphoglycerate kinase (which participate in the EMP pathway), while the rise in the amount of 6PG and the reduction in the mounts of Ribu5P and Ribo5P were indicative of the inhibition of 6-phosphogluconate dehydrogenase, an enzyme that participates in the PP pathway (Figure 2). It is be possible that fluoride inhibits various reactions in the central carbon metabolic pathways *in vivo*, although further studies are needed to confirm this.

Xylitol is a non-fermentative sugar alcohol and, thus, does not cause dental caries. Xylitol repress acid production from glucose by *Streptococcus mutans*. Specifically, glycolytic enzymes are inhibited by xylitol 5-phosphate (X5P), which can be produced from xylitol through the phosphoenolpyruvate fructose phosphotransferase system [32,50,51]. Furthermore, X5P is then dephosphorylated and converted back into xylitol, resulting in the formation of a “futile cycle”, *i.e.*, an energy-wasting cycle. As a result, xylitol inhibits the growth of *Streptococcus mutans* [52,53]. In the oral biofilm, there were no obvious changes in levels of metabolite in the presence of xylitol except for the detection of X5P (Table 2). X5P was detected at a significant level only after a rinse with a xylitol-glucose mixture (Table 3), indicating that X5P is produced via a bacterial phosphoenolpyruvate-dependent phosphotransferase system [50,53]. However, X5P does not seem to influence glucose fermentation by supragingival plaque *in vivo*, *i.e.*, lactate was produced regardless of the presence/absence of xylitol (Table 3).

The *in vivo* effects of fluoride on metabolism and lactate production in the oral biofilm were basically consistent with those previously reported *in vitro* results. In addition, the *in vivo* metabolomics profiles of the oral biofilm suggested that fluoride acts via multiple inhibitory mechanisms in the oral biofilm. However, they also indicated that xylitol cannot inhibit glucose metabolism in the oral biofilm. Thus, *in vivo* metabolomic data about the oral biofilm might provide new information that cannot be obtained from *in vitro* studies using a single bacterial species. CE-TOFMS-based metabolomic studies of the oral biofilm are expected to provide us with various insights concerning the site-specific diagnosis of oral diseases and the localized clinical effects of various medicines and nutrients on such diseases. In addition, it might even aid oral health status monitoring.

The oral biofilm is able to utilize various metabolic substrates other than glucose and, hence, produces a wide variety of metabolites. However, CE-TOFMS is not able to measure the levels of smaller (molecular weight < 50), neutral (non-ionic), or volatile metabolites, such as acetic acid, ammonia, and hydrogen sulfide, which are also produced in the oral biofilm. To enable more comprehensive metabolomics analyses, it will be necessary to combine CE-TOFMS with other techniques, such as liquid chromatography and gas chromatography, which can be used to measure the levels of small and volatile metabolites.

## 3. Application to Oral Cancer Research

Recently, cancer has also been investigated using a CE-TOFMS-based metabolomic approach [6,54,55,56,57,58]. In these studies, the cancer tissue from patients or the cultured cancer cell were used as the samples. The cancer tissue sample was collected from excited cancer tissues using sterilized tools, weighed with an electronic scale, and frozen immediately in liquid nitrogen. The frozen samples were crushed completely by a cell disruptor, then, pre-treated and analyzed similarly to the oral biofilm sample. The cultured cancer cell sample was also pre-treated similarly to oral biofilm.

In a study of colon and stomach cancer tissues by Hirayama *et al.* [6], enhanced glycolysis and lactate production (the Warburg effect) were observed in both types of cancer tissues, and significant alterations in the levels of TCA cycle metabolites were also detected. Much higher levels of citrate (CA), AC, iCA, and 2-oxoglutarate (2OG) were detected in the stomach cancer tissue than in the colon cancer tissue. Furthermore, significantly increased levels of succinate were in the colon cancer tissue, but not in the stomach cancer tissue. The levels of amino acids and their related metabolites were also measured by CE-TOFMS, and almost all of them exhibited increased levels in both the stomach and colon cancer tissue. Kami *et al.* [55] compared the metabolome profile in lung and prostate cancer tissues. In the prostate cancer tissue, almost no EMP pathway metabolites were detected. In addition, the levels of CA, AC, and iCA were increased in prostate cancer, while they were decreased in lung cancer. These observations indicate that analyses of the whole metabolic profile, e.g., the EMP pathway, the PP pathway, the TCA cycle, and amino acid metabolic pathways might help to clarify the physiological characteristics of each type of cancer [6].

**Figure 3:**
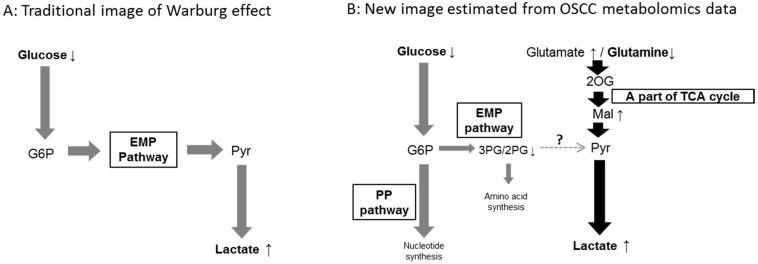
(**A**) The traditional image of the Warburg effect; and (**B**) a new concept based on metabolomic data obtained from OSCC samples. Gray bold arrows: metabolic flows derived from glucose metabolism; Black bold arrows: metabolic flows derived from glutaminolysis; Small arrows with metabolite names: a significant increase (upward arrow) or reduction (downward arrow) in the levels of the named metabolites; See Figure 1 for an explanation of the metabolite abbreviations.

Ogawa *et al.* performed metabolomics analyses of clinical specimens of oral squamous cell carcinoma (OSCC) using CE-TOFMS, and characterized their metabolic systems [59]. OSCC tissue and the surrounding normal tissue from patients with OSCC were used. Most metabolites were analyzed by CE-TOFMS, while glucose was quantified enzymatically [60]. Significant differences in the levels of some of the examined metabolites were detected between the OSCC and normal tissues (Table 4). For example, the OSCC tissue exhibited decreased levels of glucose and glutamine, and an increased level of lactate, suggesting that glucose consumption and lactate production were enhanced (the Warburg effect) and glutamine was metabolized (glutaminolysis) in the OSCC tissue. The reduction in the levels of metabolic intermediates belonging to the EMP pathway suggested that the metabolic intermediates derived from glucose are partly transferred to the PP pathway, whereas glutaminolysis may provide lactate (via the last half of the TCA cycle and pyruvate) (Figure 3). It is possible that the Warburg effect, *i.e.*, enhanced aerobic glucose consumption and lactate production, stems from the combination of enhanced glucose consumption and glutaminolysis in OSCC tissue (Figure 3).

These metabolomic approaches may offer novel insights into cancer metabolism, e.g., cancer-specific metabolites and metabolic pathways. In the future, the metabolome analysis might support diagnosing the early stage of cancer, which is difficult to be distinguished by conventional inspection.

Information about cancer type-specific metabolic pathways and the *in vivo* effects of anticancer agents on these pathways might contribute to the development of new strategies for diagnosing specific cancers and more effective anticancer agents. For example, Urakami *et al.* [56] investigated the effects of 2-deoxy-d-glucose (2DG), an anticancer agent, on endometrial cancer cell metabolism using a metabolomics-based approach. 2DG is generally considered to inhibit the glycolytic pathway by inducing the accumulation of 2-deoxy-d-glucose-6-phosphate. However, in the latter study various metabolites derived from 2DG were detected in the EMP pathway, the PP pathway, and the TCA cycle, suggesting that 2DG and its metabolites might also interfere with various biological processes and act as anti-cancer agents. These kinds of studies, especially those examining anti-cancer agents, are in their early stages, so further studies are needed to confirm their findings.

## 4. Application to Saliva Samples

In addition to studies of the oral biofilm and oral tissue, CE-TOFMS-based metabolomics studies are also useful for research into bodily fluids, including saliva. Saliva is a biological fluid derived from blood that reflects the physiological state of the body, as well as oral biofilm metabolism. In other words, both oral disease and systemic metabolic disorders diseases might induce changes in the components of saliva. Therefore, monitoring the composition of saliva might aid the diagnosis or prediction of both oral and systemic diseases. In metabolomics studies of the oral biofilm and oral cancer tissue, the data are mainly used to analyze their metabolic mechanisms and characteristics, which might be associated with the pathogenesis of various conditions. On the other hand, in metabolomics studies of saliva, the data are often used to search for disease biomarkers that would be useful for screening and diagnosis. Furthermore, collecting saliva is easy, simple, non-invasive, and inexpensive. Thus, saliva sample-based metabolomic diagnosis techniques for various conditions, including oral cancer [61,62,63], periodontitis [61], and Sjögren’s syndrome [64] have been reported. Unlike studies on cancer and oral biofilm, in saliva studies the comprehensive analysis of the wide range of metabolites is required. CE-TOFMS is a good analyzer for the metabolic intermediates in the carbon metabolism and the amino acid metabolism as stated above, while other analytical devices can target other groups of metabolites. Therefore, metabolome analysis using various analytical devices has been performed in saliva research.

Sugimoto *et al*. [61,63] tried to identify diluted, and analyzed with CE-TOFMS. They suggested that quantitative information of about 57 specific metabolite profiles for oral, breast, and pancreatic cancer and periodontal disease using saliva sample. They collect 5 mL of unstimulated saliva from subjects at 5 min after rinsing with water. The saliva collected was centrifuged enough and the supernatant was used as a sample. The sample was metabolites and their combinations can be used to predict these diseases’ susceptibility and that these metabolites might be useful biomarkers for screening. However, several limitations need to be thought about in saliva research. Sugimoto *et al*. [65] revealed that salivary metabolite profiles may have variations by many physiological and environmental factors, such as collection time, collection method, sex, BMI, and smoking. For avoiding these variations as much as possible, the authors asked subjects to refrain from eating, drinking, smoking, or using oral hygiene products for at least 1 h prior to saliva collection. Furthermore, saliva samples may include metabolites derived from both oral biofilm and oral tissues, and it is difficult to separate the metabolites to each origin. Nevertheless, this study found a positive correlation between the some metabolites and diseases.

Wang *et al.* [62] also tried to identify OSCC using saliva samples. They pretreated the saliva sample similarly, but used ultra-performance liquid chromatography (UPLC)-MS as a metabolome analysis device. They also found that the levels of 14 salivary metabolites up- or downregulated in OSCC patients. In addition, the use of five candidate biomarkers (propionylcholine, *N*-Acetyl-l-phenylalanine, sphinganine, phytosphingosine, and *S*-carboxymethyl-l-cysteine) in combination resulted in satisfactory accuracy, sensitivity, and specificity in distinguishing early-stage OSCC from the control. Kageyama *et al.* [64] tried to identify biomarkers that are useful for diagnosing primary Sjögren’s syndrome. They use gas chromatography (GC)-MS as a metabolome analysis device. The pre-treatment of sample was different to the other two studies. Collected salivary samples were centrifuged, and the supernatants were mixed with methanol, chloroform, and H_2_O, incubated for 30 min, and centrifuged again. The supernatants were oximated, derivatized, cleaned by removing debris, and analyzed with GC-MS. They found that 41 metabolites exhibited lower levels in the patients’ samples, and the salivary metabolite profile of the patients was less diverse than that of the healthy control.

These results suggest that the metabolome profile or the combination of some metabolites in saliva can be useful for detecting oral diseases, although several methodological limitations exist.

## 5. Conclusions and the Future Research

Metabolomic research into the oral biofilm, oral cancer, and saliva is in its early stages, but it has already revealed several novel findings about the properties metabolisms, that is, their physiological functions. In studies into the oral biofilm, it was shown that the metabolic processes, which were previously assessed based on studies involving a single bacterial species, are in operation in the oral biofilm, while novel findings regarding the inhibitory effects of fluoride and xylitol were obtained. These results suggest that the metabolomic research using real oral biofilm is essential for clarifying the pathogenesity of the oral biofilm. In oral cancer research, the metabolomic approach has offered various novel insights into cancer metabolism, e.g., it has identified various cancer-specific metabolic pathways. In addition, metabolomics analyses of saliva useful human diagnostic method for both oral and systemic diseases, since saliva sampling is simple, easy, non-invasive, and inexpensive. In the future, metabolomic analyses of oral specimens, such as oral biofilm, oral tissue, and saliva samples, might provide a wide range of novel information, leading to more accurate diagnosis, safer and more effective treatment, and preventive strategies of oral and systemic disorders.

## Figures and Tables

**Table 1:** The amount of metabolites before and after glucose rinse in the oral biofilm [39].

Metabolites		Concentration (nmol/mg Wet Weight of Plaque)	
		Before	After
EMP pathway			
	G6P	0.133 ± 0.032 ^#^	0.442 ± 0.087 *
	F6P	0.033 ± 0.006	0.108 ± 0.025 *
	F1,6BP	0.024 ± 0.010	0.099 ± 0.083
	DHAP	0.037 ± 0.004	0.074 ± 0.011 *
	3PG	0.245 ± 0.165	0.218 ± 0.159
	PEP	0.094 ± 0.035	0.062 ± 0.036
	Pyr	0.588 ± 0.461	4.235 ± 2.731
Lactate		1.737 ± 0.823	13.12 ± 12.71
PP pathway			
	6PG	0.008 ± 0.002	0.033 ± 0.017
	Ribu 5P	0.029 ± 0.014	0.054 ± 0.021 *
	Ribo 5P	0.011 ± 0.007	0.036 ± 0.027
	S7P	0.058 ± 0.019	0.143 ± 0.041 *
	E4P	ND	ND
TCA cycle			
	ACoA	0.020 ± 0.006	0.045 ± 0.019
	CA	0.038 ± 0.031	0.017 ± 0.006
	AC	0.001 ± 0.003	0.000 ± 0.001
	iCA	0.001 ± 0.002	0.000 ± 0.000
	2OG	0.013 ± 0.013	0.023 ± 0.012
	SCoA	0.011 ± 0.018	0.019 ± 0.022
	Suc	1.834 ± 1.320	1.650 ± 1.001
	Fum	0.034 ± 0.039	0.018 ± 0.019
	Mal	0.105 ± 0.059	0.074 ± 0.044

^#^ Mean ± standard deviation; Values are the mean of five individuals; * Significant difference from the amount before glucose rinse (*p* < 0.002, paired *t*-test); ND, not detected. See Figure 1 for abbreviations of metabolites.

**Table 2:** Effects of xylitol and fluoride on levels of each metabolite in the oral biofilm [40].

Metabolites		The Rate of Changes in Levels of Metabolites (Times)		
		Xylitol	225 ppm F	900 ppm F
**EMP pathway**				
	G6P	1.04 ± 0.56 ^#^	1.40 ± 0.32 *	1.76 ± 0.60
	F6P	0.81 ± 0.79	3.39 ± 4.78	2.18 ± 1.03
	F1,6BP	0.79 ± 0.80	1.64 ± 0.64	4.27 ± 2.70
	DHAP	0.87 ± 0.40	0.57 ± 0.45	0.44 ± 0.28
	3PG	0.94 ± 0.70	3.57 ± 2.46	9.22 ± 5.22
	PEP	0.95 ± 0.80	0.83 ± 0.83	0.57 ± 0.63
	Pyr	0.82 ± 0.59	0.41 ± 0.19	0.29 ± 0.15
Lactate		1.02 ± 0.55	0.70 ± 0.21	0.59 ± 0.31
**PP pathway**				
	6PG	0.97 ± 0.67	2.09 ± 0.99	4.40 ± 2.48
	Ribu 5P	0.91 ± 0.40	0.80 ± 0.33	0.81 ± 0.21
	Ribo 5P	0.83 ± 0.37	0.39 ± 0.45	0.28 ± 0.14
	S7P	0.92 ± 0.45	1.18 ± 0.23	2.90 ± 3.10
	E4P	ND	ND	ND

^#^ The rate of change was calculated as (amount of metabolite after glucose rinse with fluoride application or xylitol-glucose rinse)/(amount of metabolite after glucose rinse). Mean ± standard deviation. Values are the mean of seven individuals; * Significant difference from the amount before glucose rinse (*p* < 0.05); ND, It could not be calculated because the metabolite was not detected. See Figure 1 for abbreviations of metabolites. There were also no clear changes in TCA cycle.

**Table 3:** The amount of xylitol 5-phosphate in the oral biofilm after glucose with/without xylitol rinse [40].

Oral Rinse with;	Concentration (nmol/mg Wet Weight of Plaque)
Glucose	ND
Xylitol + Glucose	13.9 ± 5.2 ^#^

^#^ Mean standard deviation; ND, not detected.

**Table 4:** The levels of metabolites, which showed significant differences between oral squamous cell carcinoma (OSCC) and normal tissues [59].

Metabolites		Concentration (nmol/mg Wet Weight of Tissue)	
		Cancer	Normal
Glucose		0.84 ± 0.67 ^#,^*	1.59 ± 1.31
EMP Pathway			
	3PG	0.38 ± 0.49 *	1.64 ± 3.81
	2PG	0.06 ± 0.07 *	0.24 ± 0.54
Lactate		76.7 ± 67.1 *	51.9 ± 43.8
TCA cycle			
	Fum	0.67 ± 0.36 *	0.52 ± 0.33
	Mal	1.83 ± 1.36 *	1.32 ± 0.88
The amino acids and the metabolites related to amino acids metabolism			
	Glutamine	9.35 ± 7.53 ^$^	11.0 ± 6.63
	Glutamate	13.8 ± 10.1 *	9.92 ± 9.52
	Glycine	8.37 ± 7.94 *	6.05 ± 6.17
	Aspartate	5.00 ± 4.69 *	2.93 ± 3.54
	Proline	2.92 ± 3.29 *	2.11 ± 2.35
	Cysteine	0.21 ± 0.21 *	0.12 ± 0.14
	Hydroxyproline	0.34 ± 0.79 *	0.23 ± 0.49
	Creatine	8.48 ± 9.39 *	14.0 ± 12.9
	Creatinine	0.30 ± 0.47 *	0.30 ± 0.27
	Putrescine	0.27 ± 1.07 *	0.08 ± 0.29

^#^ Mean standard deviation; * Significant difference from the amount in normal tissue (*p* < 0.0125); ^$^ The level of glutamine in OSCC was lower than that in normal tissues, although the difference was not significant. See Figure 1 for abbreviations of metabolites.
